# Uncontrolled severe T2 asthma: Which biological to choose? A biomarker-based approach

**DOI:** 10.3389/falgy.2022.1007593

**Published:** 2022-11-14

**Authors:** Antolín López-Viña, Rocío M. Díaz Campos, Andrea Trisan Alonso, Carlos Melero Moreno

**Affiliations:** ^1^ Servicio de Neumología, Hospital Universitario Puerta de Hierro, Majadahonda, Madrid, Spain; ^2^ Servicio de Neumología, Hospital Universitario 12 de Octubre, Madrid, Spain; ^3^ Instituto de Investigación (i+ 12), Hospital Universitario 12 de Octubre, Madrid, Spain

**Keywords:** T2 severe asthma, monoclonal antibodies, biomarkers, exacerbations, systemic corticosteroid

## Abstract

In recent years, advances in knowledge of molecular mechanisms involved in asthma have changed uncontrolled severe asthma (USA) treatment, with the appearance of biological treatment. USA is a heterogeneous entity with different endotypes and phenotypes. Nowadays, the biological drugs approved with asthma indication are omalizumab, mepolizumab, reslizumab, benralizumab and dupilumab. Tezepelumab is approved by the Food and Drug Administration (FDA) in the United States and, recently, by the European Medicines Agency (EMA). All these biological drugs have shown their efficacy in clinical trials, especially in reducing exacerbations, improving asthma control, quality of life, pulmonary function, and withdrawing systemic corticosteroids or at least reducing their daily dose, with some differences between them. Except for mepolizumab and reslizumab, biological drugs have different targets and thus different therapeutic indications should be expected; however, in some patients, more than one drug could be indicated, making the election more difficult. Because there are no direct comparisons between biological drugs, some biomarkers are used to choose between them, but they are not unbeatable. In this article, an algorithm to choose the first biological drug in a specific patient is proposed based on different study results and patient’ characteristics.

## Introduction

Severe asthma (SA) requires high doses of inhaled corticosteroids (ICS) associated with another controller medication [long-acting beta 2 adrenergic agonists (LABA) and/or long-acting anticholinergic (LAMA)] to be controlled or despite that remains uncontrolled. Therapeutic adherence, comorbidities, and triggers must be evaluated before (1).

Uncontrolled severe asthma (USA) is considered when SA remains uncontrolled despite high doses of ICS and LABA or oral corticosteroids (OCS), for at least, 6 months in 1 year without any other cause than disease severity (1).

Although the USA is approximately 5% of the asthmatic population (2), it accounts for more than half of asthma costs and is responsible for daily symptoms, frequent exacerbations, and hospitalizations (3, 4). OCS adverse events (AE) must be considered (5, 6).

SA is a heterogeneous syndrome considering allergy presence, symptoms onset, airway obstruction severity, treatment response, and prognosis. Recently, SA is considered biologically heterogeneous with differentiated subtypes characterized by different pathophysiological mechanisms so endotypes or molecular phenotypes are defined (7–11). There are two endotypes: high T2 and low T2.

High T2 endotype is driven by T2 cytokines (IL-4, IL-5, and IL-13), eosinophils, alarmins [IL-25, IL-33, and thymic stromal lymphoprotein (TSLP)], and IgE. There are two groups of patients with high T2 SA: early-onset allergic asthma and late-onset eosinophil asthma (12–14).

There are different biomarkers to identify high T2 inflammation (15–18): blood and sputum eosinophils, exhaled fraction of nitric oxide (FeNO).

Clinical practice guidelines (19–21) based on different studies determine as cut-off points: blood eosinophils ≥150/µl, and/or FeNO ≥ 20ppb, and/or induced sputum (IS) eosinophils ≥2%. IgE cannot be considered a T2 biomarker, although it is used to calculate omalizumab’s dose, but an allergic prick test or specific IgE determination can.

Low T2 endotype mechanism knowledge is poor, and no biomarkers for its identification are available (22). It is characterized by neutrophilic or pauci-granulocytic inflammation (7).

Advances in knowledge of T2 asthma molecular mechanisms have led to the appearance of monoclonal antibodies (MA) which target immunoglobulins and cytokines implicated in the inflammatory cascade (23). Nowadays, biological drugs approved by regulatory agencies in the United States (FDA) and Europe (EMA) are Omalizumab (MA that blocks IgE union to its receptor for allergic asthma), mepolizumab and reslizumab (anti-IL-5 MA for eosinophilic asthma), benralizumab (antagonist of the α subunit of IL-5 receptor for eosinophilic asthma), and dupilumab (directed against the *α* subunit of IL-4 receptor which is a common receptor for IL-4 and IL-13). Tezepelumab [anti-alarmin directed against the thymic stromal lymphopoietin (TSLP)] has shown its efficacy in the USA approved by FDA, and recently, by EMA.

Clinical trials and real-world studies, with T2 USA patients, have shown MAs' efficacy in reducing exacerbations, improving asthma control, pulmonary function, and withdrawing or at least reducing OCS daily dose.

A great response variability is seen in clinical practice, from super-responders (no symptoms, no exacerbations) to sub-optimal or no responders at all. To make precision medicine, it would be ideal to know which MA is the best for each patient, but no head-to-head comparisons are available while indirect comparisons (24–28) are but with low value mainly because of arbitrarily inclusion criteria.

The best scenario would be to have predictor markers of good or bad response to MA but until they are available the MA election would be based on asthma patients’ characteristics.

This article will revise different biomarkers studies that could predict MA response in T2 asthma patients, differences in MA clinical trials, and their impact on comorbidities. Also, the importance of treatment goals for each patient, the patient’s age, and the patient’s treatment preferences with the aim to propose an algorithm to choose the first MA in a specific patient.

## Possible biomarkers

### Blood eosinophil count

Is the most frequent biomarker used to predict therapeutic response to all MA. Its determination is cheap, minimally invasive, and easy to obtain. Although it has some drawbacks as temporary fluctuations in time (29) and its reduction with some drugs mainly OCS. Repeated determinations improve its sensibility. In patients taking OCS is recommended to reduce the dose and realize different determinations (30).

Response to anti-IL5/IL5R*α* MA is better with more eosinophils in the blood.

Exacerbation reduction with mepolizumab is better with more than 300 blood eosinophils/µl and almost nil under 150 eosinophils/µl (31, 32). With reslizumab, a response is almost nil with less than 400 eosinophils/µl (33), while benralizumab’s efficacy is better with more than 300 blood eosinophils/µl (34). With dupilumab (35, 36), there is a better response with more than 300 eosinophils/µl.

EXTRA (37) *post hoc* analysis with omalizumab showed a better response with more than 300 eosinophils/µl, although this result was not seen in other studies.

Tezepelumab’s efficacy is better with more than 150 eosinophils/µl, although it has shown benefits in patients without eosinophilia (38).

### Sputum eosinophils

The “gold standard” test to diagnose T2 asthma is the presence of eosinophils in sputum. The relationship between an increase in exacerbations and more eosinophils in sputum has been demonstrated (39, 40). Some factors such as cost, availability and time, make it difficult to use this biomarker, so it is available only in some hospitals.

In clinical practice, eosinophils in sputum are not validated to choose a MA although they could be used to evaluate treatment response. Data exists about the persistence of eosinophils in sputum in non-responder patients treated with mepolizumab, indicating T2 residual inflammation, so a switch to reslizumab (41) could be an option (weight dose calculated).

### FeNO

FeNO is a non-invasive parameter of epithelial damage and bronchial T2 phenotype associated with eosinophil inflammation. It is related to different T2 citoquines, mainly with IL13.

FeNO does not predict anti-eosinophil MA (anti IL5/IL5R*α*) response. No differences are seen in patients’ responses with high or low FeNO.

Exacerbation reduction with dupilumab (36) is greater with FeNO values ≥ 25ppb so it is used as a response biomarker.

Omalizumab’s response was better with FeNO ≥ 24ppb in the EXTRA study (37), although this result was not shown in other studies.

Exacerbation reduction with tezepelumab is also predicted by FeNO values (38).

### Blood IgE

Blood IgE cannot be considered a T2 inflammation biomarker. A relationship between IgE level and atopic asthma exists, although high blood IgE levels can be seen in patients with non-atopic asthma (42).

Omalizumab’s dose is calculated considering blood IgE level but exacerbation reduction is independent of IgE values (43).

Dupilumab was effective in allergic and non-allergic asthma patients in the QUEST study, even with high blood IgE level (>700 IU/ml), so dupilumab could be used in allergic asthma with blood IgE level >1,500 IU/ml (44).

Blood IgE level does not predict response to anti-IL5 MA.

### Periostin

Periostin is a protein detected in peripheral blood and secreted by epithelial cells in response to IL4/IL13. Initially, it arose a lot of expectations as a response detector mainly for anti-IL13 MA. Periostin is associated with tissue remodeling in asthma and does not always correlate with eosinophils and other biomarkers (45). Nowadays, the interest in periostin as a response predictor has decreased.

## Differences between MA in pivotal studies

### Omalizumab

Clinical trials (46–50), meta-analysis (51), and real-world studies (52, 53) have shown Omalizumab's efficacy in ≥ 6 years old USA patients with sensitization for at least one perennial allergen and IgE blood levels from 30 to 1,500 IU/l. Exacerbations reduction, asthma control improvement, and moderate and inconsistent pulmonary function improvement have been demonstrated. Data about OCS reduction or withdrawal is contradictory (Table 1).

**Table 1:** Monoclonal antibodies’ results differences.

	Indication	Biomarkers	Primary outcomes	Secondary outcomes	OCS dose reduction/withdrawal	Dose/route of administration
Omalizumab	Allergic asthma		Exacerbations reduction/quality of life improvement	Symptoms control and pulmonary function improvement		Every 2–4 weeks/SC
Mepolizumab	Eosinophilic asthma	Blood eosinophils	Exacerbations reduction/quality of life improvement	Symptoms control, quality of life and FEV1 improvement	Yes	Every 4 weeks/SC
Reslizumab	Eosinophilic asthma	Blood eosinophils	Exacerbations reduction/FEV1 improvement	Symptoms control, quality of life and pulmonary function improvement		Every 4 weeks/IV
Benralizumab	Eosinophilic asthma	Blood eosinophils	Exacerbations reduction/FEV1 improvement	Symptoms control and quality of life improvement	Yes	Every 8 weeks (every 4 weeks the first 3 doses)/SC
Dupilumab	T2 asthma	Blood eosinophils, FeNO	Exacerbations reduction/FEV1 improvement	Symptoms control and quality of life improvement	Yes	Every 2 weeks/SC
Tezepelumab	Severe asthma	Blood eosinophils, FeNO	Exacerbations reduction	Qualitiy of life and FEV1 improvement		Every 4 weeks/SC

FeNO, exhaled fraction of nitric oxide; FEV1, forced expiratory volume in the first second; IV, intravenous; OCS, oral corticosteroids; SC, subcutaneous.

### Mepolizumab

Exacerbations reduction (54–56), OCS dose reduction (57, 58), improvement in quality of life (54, 56, 57), asthma control (56, 57), and forced expiratory volume in the first second (FEV1) (56) have been shown in clinical trials with USA patients treated with mepolizumab.

Mepolizumab’s efficacy is better with more blood eosinophils as has been shown in pivotal clinical trial extension studies (31).

### Reslizumab

Exacerbation reduction has been shown in clinical trials (59, 60) although in one (60) of them was only seen in the patient subgroup with 400 blood eosinophils/µl. Improvement in FEV1, asthma control, quality of life, and pulmonary function parameters [forced vital capacity (FVC) and forced mid-expiratory flow (FEF_25–75_)] have been shown in clinical trials (59, 61).

### Benralizumab

Clinical trials’ primary endpoints have demonstrated exacerbations reduction (62, 63) in patients with ≥300 eosinophils/µl, FEV1 improvement (64, 65), and OCS dose reduction (66).

Clinical trials’ secondary endpoints have shown asthma control (62, 63) and quality of life (67) improvement.

### Dupilumab

Exacerbations reduction (36, 38) and OCS dose reduction (35) have been shown in clinical trials’ primary endpoints.

Clinical trials’ secondary endpoints have shown FEV1, asthma control, and quality of life improvement (36, 38, 69).

Exacerbation reduction and FEV1 improvement are greater in dupilumab patients treated with ≥300 blood eosinophils/µl and FeNO ≥ 25ppb (70).

### Tezepelumab

Exacerbation reduction (38, 71) in patients with >300 blood eosinophils/µl (38) has been shown in clinical trials’ primary outcome and FEV1 and quality of life improvement (38, 71) in clinical trials’ secondary endpoints. Exacerbation reduction has been shown in patients without eosinophilia, in clinical trials, so it could be indicated in low T2 USA.

## MA impact in frequent comorbidities in asthma patients

### Omalizumab

Omalizumab’s efficacy in chronic rhinosinusitis with nasal polyps (72) was evaluated in two phase 3 clinical trials that showed endoscopic and nasal congestion improvement. It is also indicated in chronic urticaria with proven efficacy (73–76) (Table 2).

**Table 2:** Monoclonal antibodies’ efficacy in different comorbidities.

	Allergic Rhinitis	Nasal polyps	Urticaria	Atopic dermatitis	Vasculitis (EGPA)
Omalizumab	Yes	Yes	Yes		
Mepolizumab		Yes			Yes
Reslizumab					
Benralizumab					
Dupilumab		Yes		Yes	
Tezepelumab					

EGPA, eosinophilic granulomatosis with polyangiitis.

Asthma patients with allergic rhinitis treated with immunotherapy have fewer systemic reactions and more probability to obtain the maintenance dose with omalizumab (76).

### Mepolizumab

A clinical trial (77) that included patients with nasal polyps refractory to medical and surgical treatment showed polyp endoscopic reduction, nasal obstruction, and symptom improvement with mepolizumab.

Mepolizumab’s efficacy is also proven in eosinophilic granulomatosis with polyangiitis (EGPA) (78) and hypereosinophilic syndrome (79).

### Reslizumab

No clinical trials are available on efficacy in comorbidities but an open-label study showed its efficacy in reducing OCS in EGPA.

### Benralizumab

Benralizumab reduces nasal obstruction and improves smell, compared with placebo, with little impact on the quality of life in patients with nasal polyps (80).

There are few case reports about benralizumab’s use in EGPA.

### Dupilumab

Dupilumab has shown its efficacy in severe atopic dermatitis (81) and was first approved with this indication.

Three clinical trials (82) have shown dupilumab’s efficacy in patients with chronic rhinosinusitis with nasal polyps in reducing polyps' size, and improving symptoms and quality of life, mainly in those patients with asthma.

## Other factors that can influence MA choice

### Treatment aims

Therapeutic goals with MA could be different from a clinical point of view. The main objective used to be exacerbation reduction (all MA have shown their efficacy in this aspect) but other goals could be asthma control or pulmonary function improvement or reduction/withdrawal of OCS.

### Symptoms control

Dupilumab (36, 38) is the MA that best controls symptoms in clinical trials. Although, extension and real-world studies have shown an improvement with all MA measured by questionnaires (83, 84).

### Pulmonary function

Benralizumab, reslizumab and dupilumab showed greater improvement in pulmonary function in clinical trials (36, 59, 64, 65, 68).

### OCS reduction

Not all the MA have shown their efficacy in reducing/withdrawing OCS. Mepolizumab, benralizumab and dupilumab have shown their efficacy in this aspect in clinical trials and real-world studies (36, 57, 58, 66).

### Possible AE

No remarkable AE has been described with any MA except injection site reactions, headache and general malaise. Dupilumab can produce transitory peripheral eosinophilia (69) (4%–13%).

### Patient’s age

Mepolizumab is indicated in USA patients ≥6 years old, dupilumab ≥12 years old, benralizumab and reslizumab ≥18 years old.

### Patients’ choice

Patients’ choice (85) always must be considered taking into account administration frequency, route of administration, and AE.

### Pregnancy

Omalizumab (86) is the only MA with studies that showed no complications during pregnancy.

## Algorithm to choose the best MA in a specific patient

Once the MA indication is done, after we have proven that uncontrolled asthma or OCS need is due to disease severity (20, 21), we have to identify if the patient has an allergic or late-onset eosinophilic asthma because many MA can be used in both cases, and sometimes they can be overlapped, which make more difficult the MA choice.

**Figure 1:**
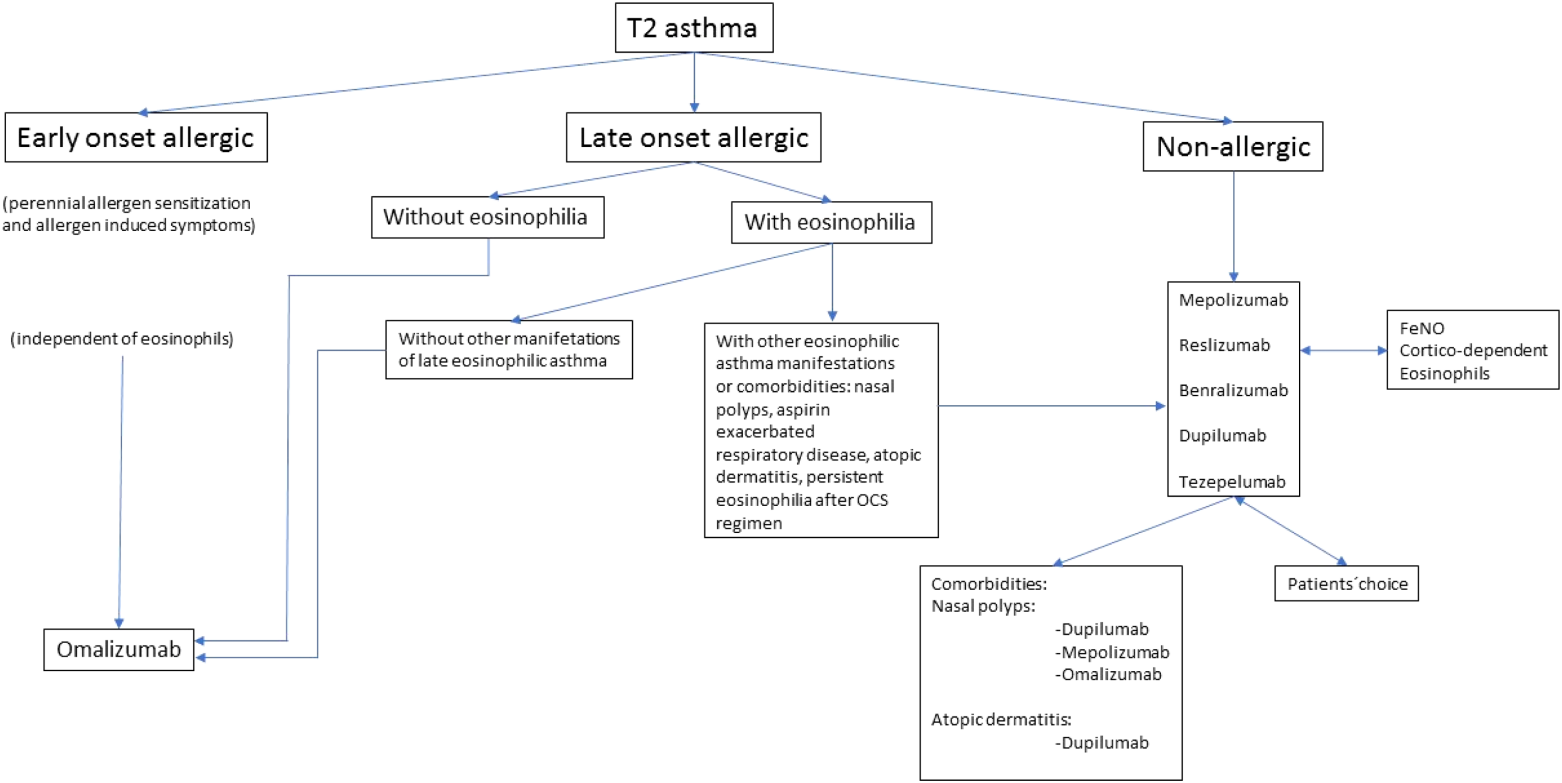
Algorithm to choose a monoclonal antibody.

We propose an algorithm for the MA choice considering clinical characteristics and biomarkers in a specific patient (Figure 1).

In clinical practice, we will find scenarios where the choice of the MA will be easy. In an early onset allergic USA patient, independently of blood eosinophil count, omalizumab will be the first choice while in a late onset non-allergic USA patient with eosinophilia, an anti-IL5/IL5Rα, or dupilumab will be considered.

In early-onset allergic asthma with eosinophilia could be used an anti-IL5/IL5R*α*, dupilumab or tezepelumab, but the main reason to choose in first place omalizumab is more years of experience. Early onset and allergy-induced symptoms predict a good response to omalizumab (87).

In our opinion, in late-onset allergic asthma patients (with perennial allergen sensitization and associated symptoms) without eosinophilia, omalizumab is the MA of choice. Although, tezepelumab could be considered.

In late-onset allergic USA patients with eosinophilia, the choice is more difficult. In this case, we must evaluate which factor have more impact on asthma control: allergy or eosinophilia. If no other late-onset eosinophilic manifestations are present, omalizumab is the MA of choice. When other eosinophilic asthma manifestations exist (nasal polyps, aspirin-exacerbated respiratory disease [AERD], atopic dermatitis or if eosinophilia persists after a short term of OCS) (88), anti IL5/IL5R*α* or dupilumab would be recommended.

In non-allergic asthma, omalizumab is not indicated, while an anti-IL5/IL5R*α*, dupilumab, or tezepelumab could be considered. No head-to-head studies are available, so we must consider the response predictor factors evaluated in clinical trials, meta-analysis, *post hoc* and real-world studies. In many cases, it will not be possible to make a high recommendation for a specific MA but different factors, described previously in this article, such as blood eosinophil count, FeNO, OCS use and comorbidities should be considered in the choice of the best MA.

Another factor to be considered is the patient’s age. Mepolizumab is indicated in USA patients ≥6 years old, dupilumab ≥12 years old, benralizumab and reslizumab ≥18 years old.

Local availability is another factor to consider. In some communities and hospitals, all the MA are not available.

Patient’s choice is the most important factor in many cases once MA’s frequency, route of administration, self-administration possibility and AE are explained.

Data from clinical trials and real-world studies showed that many patients had a good response to MA. But a good response is not always obtained, so a close follow-up must be performed mainly looking for exacerbations reduction, OCS reduction/withdrawal, symptoms, and pulmonary function improvement. A tool that combines all these parameters have been proposed (89) and may be of interest in clinical practice. If a good response is not obtained, a MA switch is recommended, but previously other causes of non-response must be ruled out such as poor adherence to ICS.

## Discussion

Monoclonal antibodies’ efficacy indicated in T2 USA has been demonstrated in clinical trials. Real-world studies’ results corroborated, even improved, them. But no head-to-head MA comparisons are available so evidence-based MA’s choice is not possible.

Clinical trials have similarities and differences that can be helpful in MA’s choice, but with poor evidence as they included different populations and used different methodologies.

Considering all these aspects, MA’s choice must take into account the different biomarkers available and patients’ characteristics.

USA included late-onset allergic and eosinophilic asthma with different mechanisms and different therapeutic targets. The first step in MA’s choice is to know the patient’s type of T2 severe asthma but sometimes it is difficult because there is an overlap of the two subtypes.

Clinical practice guidelines (19–21) and experts’ opinions (18, 83, 84, 88, 90) proposed algorithms to choose MA based on biomarkers mainly FeNO and blood or sputum eosinophil count. The algorithm we propose in this article tries to guide the MA election in each case as we do in clinical practice. Sometimes the MA election will not be the best and we will have to switch the MA.

The first step in our algorithm is to classify the patient according to the age of asthma onset and allergy presence, then eosinophilia presence must be considered.

Omalizumab would be indicated in early and late-onset allergic asthma without eosinophilia (although other MA could be chosen) mainly based on years of experience compared with other MA.

Late-onset allergic asthma with eosinophilia, represents the T2 subtypes overlap. In this case must be considered the factor that mainly influences asthma control (allergy presence or eosinophilia) evaluating the presence of other characteristics or comorbidities of late-onset eosinophilic asthma such as nasal polyps, atopic dermatitis, aspirin-exacerbated respiratory disease. Omalizumab would be indicated if these characteristics are not present but if they are, MA for eosinophilic asthma would be considered. Oishi et al. (88) proposed in overlapped patients (allergic and eosinophilic) to indicate a short OCS regimen and evaluate eosinophil response. If eosinophils disappeared, allergy probably would be the most important factor, and omalizumab would be indicated.

MA election in USA non-allergic T2 is a multifactorial process based on biomarkers, steroid dependence, and comorbidities. In our opinion, in clinical practice, generally is easy to choose a MA. Other factors to consider are MA availability and patients' preferences.

The main weakness of the algorithm we propose is that is based in our opinion and clinical experience as no head-to-head MA comparisons are available.

In conclusion, in USA is important to choose the best MA in each patient although sometimes is difficult as no direct comparisons are available and all recommendations are based on experts' opinions. MA election must be based on multiple factors including patients’ preferences.

## Data Availability

The original contributions presented in the study are included in the article/Supplementary Material, further inquiries can be directed to the corresponding author/s.
