# Corrigendum: Uncontrolled severe T2 asthma: Which biological to choose? A biomarker-based approach

**DOI:** 10.3389/falgy.2022.1119941

**Published:** 2023-01-10

**Authors:** Antolín López-Viña, Rocio M. Diaz Campos, Andrea Trisan Alonso, Carlos Melero Moreno

**Affiliations:** ^1^ Hospital Universitario Puerta de Hierro Majadahonda, Madrid, Spain; ^2^ Hospital Universitario 12 de Octubre, Madrid, Spain; ^3^ Fundación Investigación Biomédica Hospital 12 de Octubre, Madrid, Spain

**Keywords:** T2 severe asthma, monoclonal antibodies, biomarkers, exacerbations, systemic corticosteroids

A Corrigendum on Uncontrolled severe T2 asthma: Which biological to choose? A biomarker-based approach By López-Viña A, Díaz Campos RM, Trisan Alonso A and Melero Moreno C. (2022) Front. Allergy 3:1007593. doi:10.3389/falgy.2022.1007593

**Error in Figure/Table**

In the published article, there was an error in Figure 1 as published. Many words in Figure 1 appeared underlined**.** The corrected Figure 1 and its caption appear below.

**Figure 1:**
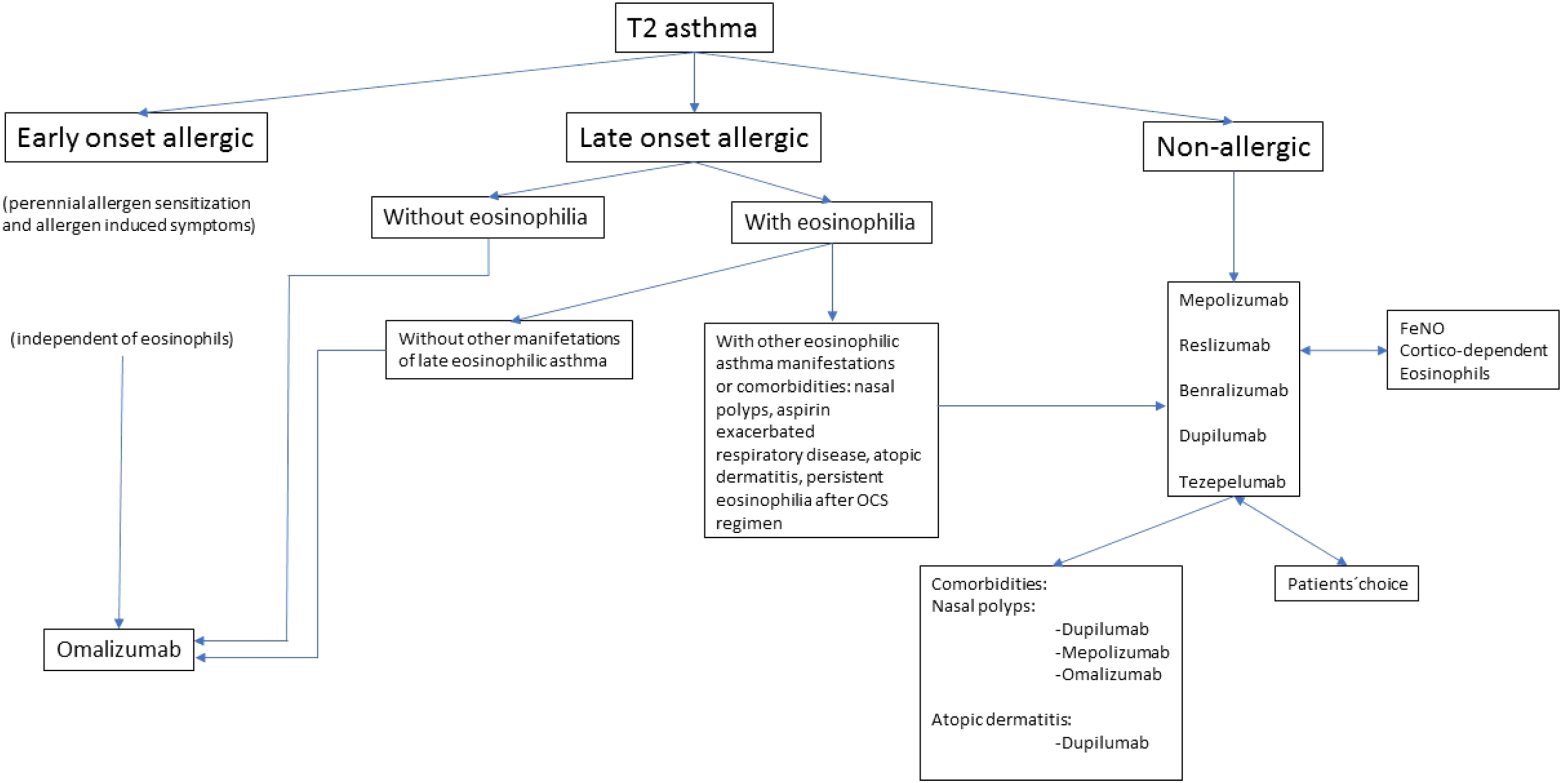
Algorithm to choose a monoclonal antibody.

The authors apologize for this error and state that this does not change the scientific conclusions of the article in any way. The original article has been updated.

